# Cardiopulmonary exercise response at high altitude in patients with congenital heart disease: a systematic review and meta-analysis

**DOI:** 10.3389/fcvm.2024.1454680

**Published:** 2024-12-24

**Authors:** Marco Vecchiato, Federica Duregon, Nicola Borasio, Sara Faggian, Veronica Bassanello, Andrea Aghi, Stefano Palermi, Gino Degano, Francesca Battista, Andrea Ermolao, Daniel Neunhaeuserer

**Affiliations:** ^1^ Department of Medicine DIMED, University of Padua, Padua, Italy; ^2^ Sports and Exercise Medicine Division, University Hospital of Padova, Padova, Italy; ^3^ Institute of Mountain Emergency Medicine, Eurac Research, Bolzano, Italy; ^4^ Independent Researcher, Padova, Italy; ^5^ Public Health Department, University of Naples Federico II, Naples, Italy

**Keywords:** congenital heart disease, cardiopulmonary exercise testing, physiological adaptations, high altitude, mountain, hiking, physical activity

## Abstract

**Background:**

An increasing number of patients with congenital heart disease (CHD) engage in physical activities and may exercise at high altitudes (HA). The physiological adaptations required at HA and their implications on individuals with CHD, especially during exercise, remain underexplored. This systematic review aims to investigate cardiopulmonary exercise responses to short-term HA exposure in individuals with CHD.

**Methods:**

A literature search was performed across PubMed, Cochrane Library, Scopus, Embase, and SPORTDiscus. The search focused on studies comparing patients with CHD to healthy controls, specifically assessing cardiorespiratory responses during cardiopulmonary exercise testing at HA (≥2,500 m) and low altitude (LA). A meta-analysis of the differences in the main cardiorespiratory adaptations during exercise from LA to HA was performed, comparing patients with CHD and controls.

**Results:**

Of the initial 4,500 articles, four studies met the inclusion criteria, encompassing 150 participants (74 with CHD and 76 controls). Almost all the patients with CHD had lower cardiorespiratory fitness and efficiency both at LA and HA compared to the controls. Nevertheless, the patients with CHD showed a smaller decrease in peak workload [10.61 W (95% CI: 2.33–18.88)] and peak saturation [1.22% (95% CI: 0.14–2.30)] between LA and HA compared to the controls. No participants presented exercise-induced symptoms.

**Conclusion:**

Short-term exposure to HA appears to be relatively well-tolerated by individuals with low-risk CHD, without a significantly different impact on cardiorespiratory response compared to healthy controls. Further research should confirm these outcomes and explore the long-term effects of higher altitude exposure as comprehensive recommendations for these patients are lacking.

## 1 Introduction

Congenital heart disease (CHD) is a leading cause of infant morbidity and mortality worldwide, with an estimated incidence of 8–10 per 1,000 live births (1). In recent years, improvements in medical and surgical treatments have led to a substantial increase in life expectancy for patients with CHD (2). This population of young and adult individuals with CHD thus has specific needs and requirements similar to those of the general population, such as traveling and exercising safely. Certain outdoor holiday destinations are characterized by high altitude (HA) and mountain regions are easily accessible and particularly attractive for recreational physical activity. HA is defined as an elevation of at least 2,500 m above sea level. Exposure and exercise at HA cause physiological changes to adapt to the reduced oxygen availability due to hypobaric hypoxia (3). These cardiac, respiratory, and hematological adaptations aim to deliver sufficient oxygen to meet the metabolic demands of the tissues (4–6).

Acute exposure to HA during air travel or mountain stays seems to be safe for most patients with CHD but the physiological changes that occur during physical exercise at HA are poorly studied (7, 8). These adaptations may pose significant challenges to physical activity at HA compared to low altitude (LA) (9). Moreover, fear regarding one’s own physical condition could limit the enjoyment and benefits of exercising in the mountains (10–12). Despite the increasing prevalence of CHD and the growing number of people living or engaging in exercise at HA, there is still limited research on this topic regarding safety guidance for exercise or evidence-based recommendations.

Therefore, the aim of this review is to summarize and discuss the current evidence on the impact and risks of acute exercise interventions during short-term exposure to HA on individuals with CHD.

## 2 Materials and methods

This is a systematic review and meta-analysis of the literature comparing the cardiopulmonary response to exercise in patients with CHD and healthy controls exposed to LA and HA.

### 2.1 Literature search

The literature search was performed in February 2024. The search strategy used in the databases was “(altitude OR mountain OR hypoxia) AND (“congenital heart disease*” OR “congenital heart defect*” OR “tetralogy of Fallot” OR “Fontan” OR “univentricular heart” OR “transposition” OR “coarctation” OR “pulmonary stenosis” OR “Ebstein” OR “Septal defect*”)”.

The research was conducted in the online PubMed, Cochrane Library, Scopus, Embase and SPORTDiscus databases. In addition, the references were examined in each eligible article, and further relevant manuscripts were screened when a positive match was observed. The search was limited to English language articles.

### 2.2 Eligibility criteria

To be included, articles needed to meet the following PICO criteria: (P) The population under investigation consisted of patients with CHD (a patent foramen ovale was considered an anatomical variant and thus not included as CHD for the purposes of this study); (I) Maximal cardiopulmonary exercise testing (CPET) must have been performed at LA and HA. HA was defined as an altitude ≥2,500 m that could be either real or simulated; (C) Each study must have a control sample of participants without CHD; (O) They should clearly define the cardiopulmonary parameters obtained during exercise.

All individuals were considered, regardless of race, ethnicity, sex characteristics, and health status. Reviews, commentaries, editorials, case reports, and letters to the editors were excluded, while published abstracts, dissertation materials, or conference presentations were not considered eligible documents.

### 2.3 Data extraction and selection process

Two investigators (FD and VB) independently examined the retrieved articles’ titles and abstracts to determine their eligibility for full-text assessment. Independent search outputs were then combined, compared, and reviewed for applicability. The review process was repeated in the case of discrepancies, and a third researcher (NB) was consulted. The selection process was conducted using Rayyan, a standardized dedicated web-app software for systematic reviews (13).

### 2.4 Quality assessment

Two investigators (FD and NB) performed the risk of bias and quality assessments; any disagreement was resolved through discussion. The Risk of Bias In Non-randomized Studies of Interventions (ROBINS-I) tool was used to analyze the risk of bias across seven domains. The studies were classified as “low,” “moderate,” “serious,” or “critical” risk of bias (14). To evaluate the quality of the included studies, the Newcastle–Ottawa Scale for case–control studies was used. Good quality studies are defined as those with three or four stars in the selection domain, one or two stars in the comparability domain, and two or three stars in the outcome/exposure domain (15).

### 2.5 Statistical analyses

The continuous variables were reported as means with standard deviations (SDs). When data were reported as the median and interquartile range (IQR), means and SDs were approximately estimated by the method described by Wan et al. (16) Heterogeneity among the studies was assessed using Cochran's Q test and the I-squared statistic. As such, a random-effects model was applied to estimate the pooled effect size and its 95% confidence interval. Pooled estimates were obtained using the mean difference (MD) in the change scores of the CPET parameters of the healthy controls and the CHD group. The change scores were calculated as the difference in each CPET parameter between LA and HA. A subgroup analysis was also specifically performed for patients with Fontan circulation as the most representative sample. Any parameters not common to all studies were not meta-analyzed. The results are shown in forest plots, with 95% confidence intervals. The online tool MA-cont was used for the meta-analysis (17, 18).

## 3 Results

### 3.1 Study characteristics

**Figure 1:**
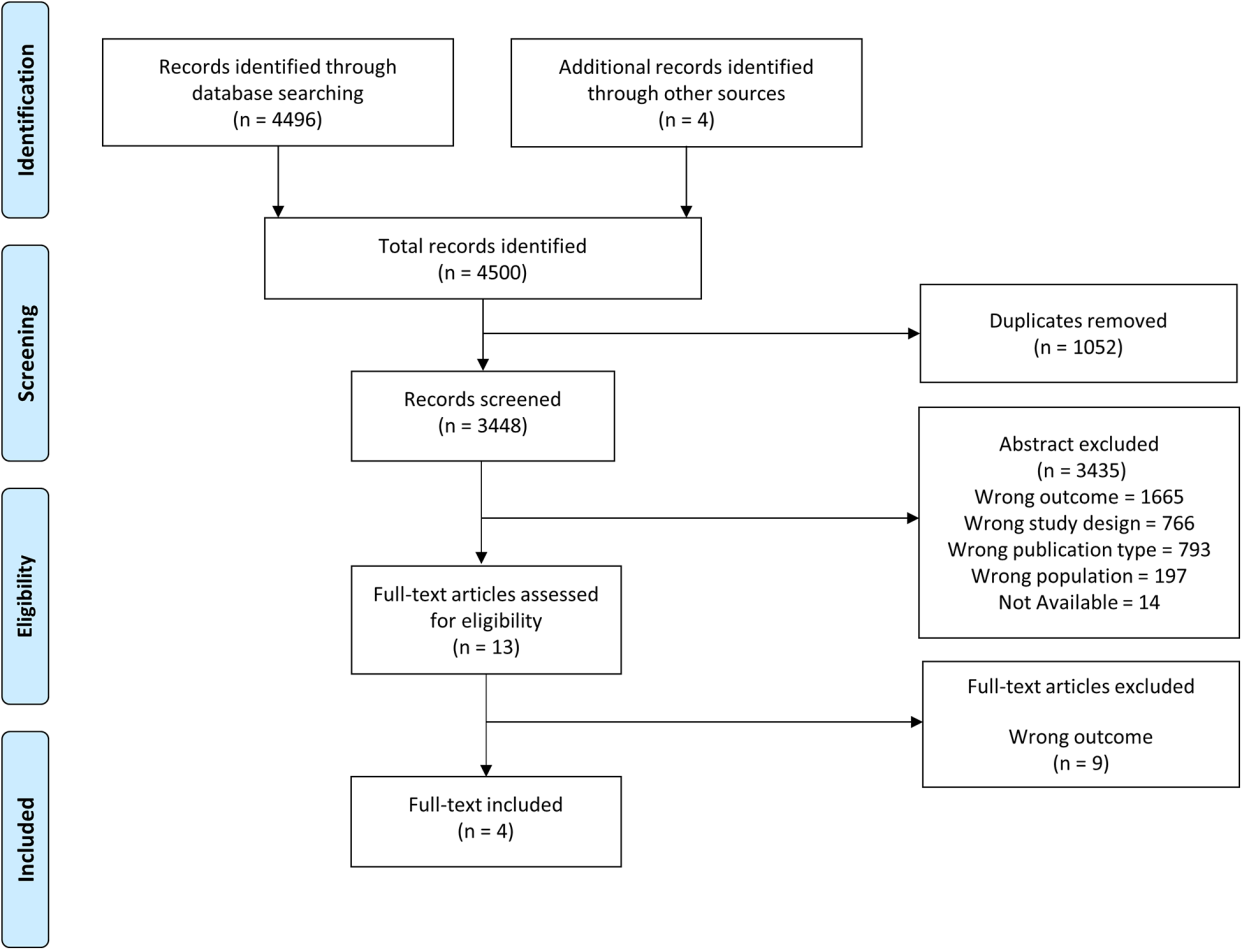
Preferred Reporting Items for Systematic Reviews and Meta-Analysis (PRISMA) flow diagram.

The flow diagram of this systematic review is presented in Figure 1, which led to four eligible studies which are shown in Table 1. A total of 150 participants were included, of whom 74 were patients with CHD and 76 were healthy controls (19–22). The median (range) total sample size of the included studies was 39 (30–42) participants. In total, 65 participants were women (43%), of whom 34 were patients with CHD (46%). The mean age of the participants with CHD and controls was 20.1 ± 5.3 and 20.4 ± 6.1 years, respectively. Three studies included only participants who had undergone the Fontan procedure (19, 20, 22) and the study conducted by Minder et al. analyzed adolescents with mixed CHD without cyanosis (21). All the included patients were in New York Heart Association (NYHA) class I or II.

**Table 1:** Characteristics of the studies.

Author	Participants (*n*)	NYHA class	Age (years)	Sex (M/F)	Altitude (m)	Exposure time at HA	Study protocol
Staempfli et al. (19)	Fontan (n = 16)	9 I/7 II	27 ± 7	6M/10F	540/3,454	6 h. Tests within 1 to 6 h	All the participants underwent two CPETs on a cycle ergometer at LA. 1st CPET: maximal test with ramp protocol. 2nd CPET: submaximal (90% of workload at RCP) test with PBF measurements with non-invasive inert gas rebreathing. The tests were repeated at HA within 12 weeks.
	Controls (n = 14)		29 ± 8	6M/8F			
Takken et al. (20)	Fontan (n = 21)	13 I/8 II	19.8 (8–37)	13M/8F	6/simulated 2,500	Duration of the test	CPET on a cycle ergometer with a ramp protocol until exhaustion, combined with non-invasive impedance cardiac output measurements, both at LA and within 3 months at simulated HA in a normobaric hypoxic tent.
	Controls (n = 21)		20.8 (8–40)	15M/6F			
Minder et al. (21)	CHD (n = 16) VSD (2) VSD-patch (1) ASD suture + VSD-patch (1) AVS (1) Repaired AVS (2) PVS (3) BAV (1) CoA (2) Repaired COA (2) TGA-arterial switch operation (1)	I–II	14.6 ± 1.2	*9M/7F*	540/3,454	5 h. Tests within 1 to 3 h	CPET on a cycle ergometer with a ramp protocol until exhaustion, PBF measurements with non-invasive inert gas rebreathing at a fixed workload below the RCP, both at LA and within 4 weeks at HA.
	Controls (n = 21)		14.8 ± 1.0	13M/8F			
Müller et al. (22)	Fontan (n = 21)	I–II	18 (14–31)	12M/9F	60/simulated 2,500	45 min. Test after 30 min of adaptation	CPET on a cycle ergometer with a ramp protocol until exhaustion combined with non-invasive impedance cardiac output measurements, both at LA and after 2 weeks at simulated HA in a normobaric hypoxic chamber.
	Controls (n = 20)		18 (14–33)	11M/9F			

F, female; M, male; mt, meters; LA, low altitude; HA, high altitude; CPET, cardiopulmonary exercise testing; RCP, respiratory compensation point; PBF, pulmonary blood flow; VSD, ventricular septal defect; ASD, atrial septal defect; AVS, aortic/subaortic valve stenosis; PVS, pulmonary valve stenosis; BAV, bicuspid aortic valve; CoA, coarctation of the aorta; TGA, transposition of the great arteries.

Two studies examined participants at real HA conditions, both at 3,454 m (Jungfraujoch, Switzerland) (19, 21), while the remaining two studies simulated HA conditions, both at an altitude of 2,500 m (20, 22). The two HA-simulating studies were conducted using a fully climatized normobaric altitude chamber and a hypoxic tent, both filled with a gas mixture of 15% oxygen and 85% nitrogen (20, 22). In the studies conducted at real HA, the LA assessment was carried out at 540 m (Bern, Switzerland). The reported length of the HA stays ranged from 45 min to 6 h. One study did not specify the duration of HA exposure (20). All CPETs were performed on cycle ergometers with a ramp protocol to reach patient exhaustion. The mean time range between CPETs at LA and HA was 7.5 (2–12) weeks (19–22). Both real HA studies specified acclimatization times before CPET (1–3 h) (19, 21). Only one of the two tests at simulated HA conditions described 30 min for adaptation to hypoxic conditions (22).

### 3.2 Study findings

The cardiopulmonary exercise test outcomes at LA and HA are listed in Table 2.

**Table 2:** Cardiopulmonary exercise test outcomes at LA and HA.

Author	Participants	Altitude	SpO_2_rest (%)	SpO_2_peak (%)	HR rest (bpm)	HR peak (bpm)	BP rest (mmHg)	BP peak (mmHg)	Workload peak (W)	VO_2_ peak (ml/kg/min)	VE/VCO_2_slope	VE peak (l/min)	SV (ml)
Staempfli et al. (19)	Patients with CHD	LA	90 ± 4	86 ± 5	84 (79–91)	172 (131–178)	—	—	118 ± 31	22.8 ± 5.1	40 ± 7	67 ± 14	Submax 52 ± 12
		HA	81 ± ± 5^a^	75 ± 5^a^	86 (78–94)	164 (132–172)^a^	—	—	99 ± 26^a^	20.5 ± 3.8^a^	56 ± 10	72 ± 16	Submax 56 ± 12
		Δ	9 ± 2 (10.0%)	11 ± 5 (12.8%)	−1 ± 8 (−2.4%)	6 ± 11 (4.6%)	—	—	19 ± 16 (16.1%)	2.3 ± 3.5 (10.1%)	−16 ± 5 (−40.0%)	−6 ± 13 (−8.9%)	/
	Controls	LA	96 (96–97)	95 (92–97)	79 ± 12	182 ± 13	—	—	194 ± 64	35.0 ± 7.4^b^	32 ± 3^b^	88 ± 27	Submax 82 ± 16^b^
		HA	87 (85–89)	81 (78–84)	81 ± 11	177 ± 13^a^	—	—	169 ± 54^a^	29.1 ± 6.5^a^^,^^b^	42 ± 4^b^	93 ± 36	Submax 87 ± 14^b^
		Δ	10 ± 3 (10.4%)	14 ± 5 (14.7%)	−2 ± 3 (−2.5%)	5 ± 14 (2.7%)	—	—	26 ± 16 (13.4%)	5.9 ± 2.8^b^ (16.9%)	−10 ± 5^b^ (−31.3%)	−5 ± 14 (−5.7%)	/
Takken et al. (20)^c^	Patients with CHD	LA	94.6 ± 2.9	93.7 ± 2.9	85.0 ± 12.3	146.9 ± 27.3	S 129.9 ± 15.8 D 73.3 ± 11.1	—	129.7 ± 44.5	1,444 ± 500 ml/min	33.6 ± 5.4	64.7 ± 22.0	Peak 80.7 ± 24.6
		HA	92.3 ± 3.5^a^	87.2 ± 4.2^a^	86.5 ± 14.3	146.0 ± 29.3	—	—	119.6 ± 41.3^a^	1,300 ± 400 ml/min^a^	38.0 ± 9.0^a^	67.3 ± 26.0	Peak 81.7 ± 31.9
		Δ	2.3%	6.9%	−1.8%	0.6%			0.6%	7.1%	−13.1%	−4%	1.2%
	Controls	LA	99.1 ± 2.1^b^	98.4 ± 1.5^b^	83.0 ± 9.8	184.9 ± 10.1^b^	S 131.2 ± 14.4 D 70.0 ± 13.1	—	255.6 ± 102.5^b^	2,800 ± 120 ml/min^b^	25.6 ± 3.7^b^	112.5 ± 48.0^b^	Peak 100.8 ± 23.1^b^
		HA	95.0 ± 3.5	90.3 ± 4.2	85.6 ± 11.7	185.6 ± 8.5	—	—	237.5 ± 93.9	2,400 ± 900 ml/min	28.9 ± 7.1	108.8 ± 39.3	Peak 107.7 ± 29.8
		Δ	4.1%^b^	8.2%	−3.1%	−0.4%	—	—	7.1%	14.3%^b^	−12.9%	3.3%	6.9%
Minder et al. (21)	Patients with CHD	LA	—	94.8 ± 1.3	64.6 ± 7.9	179.4 ± 13.1	S 113.1 ± 16 D 58 ± 61	S 127 ± 22	164.1 ± 52.2	40.2 ± 9.2	28.7 ± 3.0	75.4 ± 18.6	Submax SV 80 ± 18
		HA	—	90.8 ± 0.9^a^	80.2 ± 8.8^a^	185.3 ± 11.2^a^	S 116.8 ± 15.6 D 60.4 ± 8.3	S 132 ± 28	144.1 ± 41.8^a^	35.9 ± 6.7^a^	36.2 ± 4.3^a^	82.9 ± 15.3^a^	Submax SV 77 ± 20
		Δ	—	4%	—	—	—	—	12%	11%	/	/	/
	Controls	LA	—	95.6 ± 0.8	67.5 ± 9.3	188.8 ± 10.4^b^	S 109.6 ± 8.0 D 56 ± 5	S 121 ± 16	199.7 ± 39.3^b^	43.7 ± 7.5	28.0 ± 3.5	92.8 ± 22.9^b^	Submax SV 81 ± 19
		HA	—	91.6 ± 1.5^a^	79.0 ± 15.4^a^	189.6 ± 8.8	S 113.2 ± 8 D 60 ± 61^a^	S 125 ± 13	157.9 ± 40.4	35.4 ± 6.7^a^	36.6 ± 4.9^a^	89.4 ± 27.3	Submax SV 79 ± 20
		Δ	—	4%	—	—	—	—	21%^b^	19%	—	—	—
Müller et al. (22)^c^	CHDs	LA	92 ± 4	89 ± 5	77 ± 12	147 ± 29	S 114 ± 10 D 75 ± 11	—	112.7 ± 31.86	25.1 ± 4.49	38.23 ± 4.35	BF 37 ± 6 b/min Vt 1.66 ± 0.46 L	Peak 71.5 ± 19.05
		HA	87 ± 5^a^	83 ± 7^a^	69 ± 13	145 ± 27	S 114 ± 14 D 72 ± 13	—	104.2 ± 29.8	22.71 ± 3.66	43.01 ± 4.76	BF 39 ± 6 b/min Vt 1.64 ± 0.47 L	Peak 77.76 ± 18.64
		Δ	5.4%	6.8%	10.4%	1.4%	—	—	7.5%	9.5%	−12.5%	—	−8.8%
	Controls	LA	99 ± 1^b^	97 ± 2^b^	79 ± 10	187 ± 13^b^	S 124 ± 13^b^ D 76 ± 11	—	209 ± 52.32^b^	39.75 ± 9.6^b^	30.73 ± 4.19^b^	BF 40 ± 8 b/min Vt 2.24 ± 0.72 L^b^	Peak 97.35 ± 28.67^b^
		HA	94 ± 2^b^	90 ± 3^b^	70 ± 10	185 ± 10^b^	S 123 ± 13 D 73 ± 9	—	194.9 ± 45.72^b^	36.6 ± 8^b^	35.55 ± 4.07^b^	BF 42.1 ± 8 b/min Vt 2.3 ± 0.73 L^b^	Peak 92.2 ± 21.75
		Δ	5.1%	7.2%	11.4%	1.1%	—	—	6.7%	7.9%	−15.7%		5.3%^b^

CHD, congenital heart disease; F, female; M, male; LA, low altitude; HA, high altitude; SpO_2_, peripheral oxygen saturation; HR, heart rate; BP, blood pressure; S, systolic; D, diastolic; VO_2_peak, oxygen consumption; VE, ventilation; Vt, tidal volume, BF, breath frequency; SV, stroke volume.

Δ is the difference between the values at LA and HA. All the values described are those reported in the studies. Only percentage Δ values for the study by Staempli et al. have been calculated and reported alongside absolute values.

^a^ Statistically significant differences within group LA-HA (*p* < 0.05).

^b^ Statistically significant differences between the patients with CHD and the control group (*p* < 0.05).

^c^ Did not provide information on the statistical differences within the control group.

#### 3.2.1 Exercise capacity

**Figure 2:**
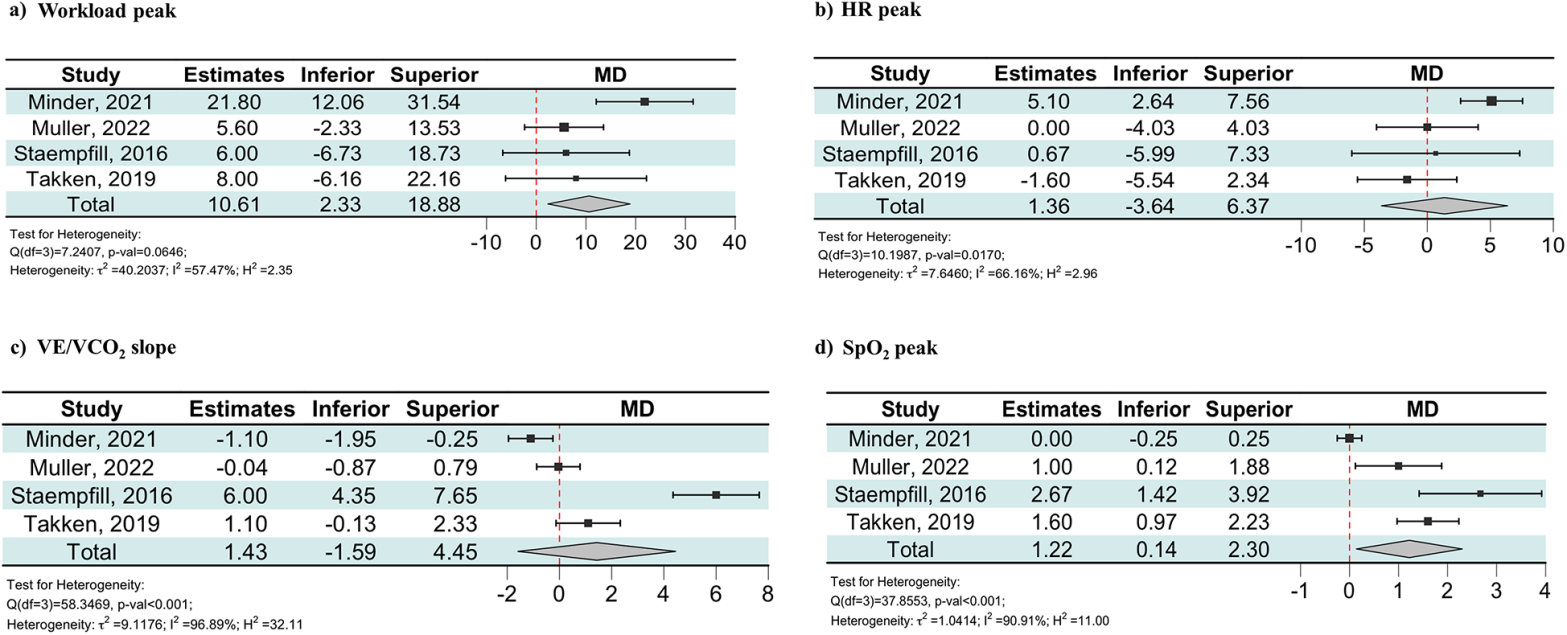
Forest plot showing the effect of maximal exercise at HA on workload peak **(a)**, HR peak **(b)**, VE/VCO_2_ slope **(c)**, and SpO_2_ peak **(d)** in the patients with CHD and the controls. Estimates were obtained using the MD of the change scores for the CPET parameters of healthy controls and the CHD group. The change scores were calculated as the difference in each CPET parameter between LA and HA. CPET, cardiopulmonary exercise test; HA, high altitude; HR, heart rate; MD, mean difference; Peak, values during peak exercise; SpO_2_, peripheral oxygen saturation; VE/VCO_2_, carbon dioxide equivalents.

The included outcomes were peak workload and peak oxygen uptake (VO_2_ peak). As shown in Figure 2A, patients with CHD showed a lower decrease in peak workload between LA and HA compared to the controls [MD: 10.61 W (95% CI: 2.33–18.88)]. When the analysis was restricted to patients with Fontan circulation, the decrease in peak workload between LA and HA remained lower compared to the controls [MD: 6.13 W (95% CI: 0.05–12.21), Supplementary Figure S1A].

Moreover, these three studies showed significantly lower values in VO_2_ peak in the patients with CHD compared to the controls, both at LA and HA (19, 20, 22). However, two studies reported a greater VO_2_ peak decrease after HA exposure in the controls than in the patients with CHD (19, 20).

#### 3.2.2 Heart rate

The included outcomes were resting and peak heart rate (HR). Only one study found a significantly higher resting HR at HA than at LA in both the CHD and control groups (21). There was no evidence of a difference from LA to HA in peak HR between patients with CHD and the controls [MD: 1.36 bpm (95% CI: −3.64 to 6.37), Figure 2B].

#### 3.2.3 Blood pressure

The included outcomes were resting and peak exercise blood pressure (BP). One study did not report BP data (19), while another provided only resting BP at LA (20). Only two studies measured resting BP at HA, showing that systolic BP was lower in the patients with CHD compared to the controls both at LA and HA (21, 22). Only one study reported systolic BP during exercise at HA, without showing any significant differences between groups (21).

#### 3.2.4 Stroke volume

All the studies analyzed stroke volume (SV) during exercise. Two studies examined SV during submaximal exercise using an inert gas rebreathing system, while the other two examined SV at peak exercise using a thoracic impedance method. In the three studies analyzing patients with Fontan circulation, the CHD group presented lower SV at LA compared to the controls (19, 20, 22). At HA, two studies reported higher SV in controls (19, 20). The two studies examining the difference in peak SV between LA and HA exposure reported conflicting results: one study reported a significant reduction in peak SV in controls compared to patients with CHD at HA, while the remaining study showed no significant differences between groups (20, 22).

#### 3.2.5 Ventilation

The included outcomes were resting and peak ventilation (VE) and respective carbon dioxide equivalent (VE/VCO_2_) slopes. Three studies reported VE as absolute values (l/min) (19–21), while the remaining provided the respiratory rate and tidal volume (22). None of the studies reported any differences in resting VE between groups at either LA or HA. Three studies showed significantly higher VE during peak exercise at LA in the control groups compared to patients with CHD (20–22). At HA, the three studies analyzing patients with Fontan circulation did not show any differences in VE modification during peak exercise between patients with CHD and the controls (19, 20, 22). Moreover, the average VE/VCO_2_ slope values were higher in patients with Fontan circulation compared to the controls at both LA and HA. Even so, as shown in Figure 2C, there was no evidence of a difference from LA to HA in VE/VCO_2_ slope modification between patients with CHD and the controls [MD: 1.43 (95% CI: −1.59 to 4.45)]. When the analysis was restricted to the three studies on patients with Fontan circulation, the change in the VE/VCO₂ slope between LA and HA remained comparable between the CHD group and the controls [MD: 2.30 (95% CI: −1.29 to 5.89), Supplementary Figure S1C]. One of these studies reported a VE/VCO_2_ slope in patients with CHD at HA of 56 ± 10.

#### 3.2.6 Peripheral oxygen saturation

Peripheral oxygen saturation (SpO_2_) was provided in resting conditions and during peak exercise. One study did not report SpO_2_ at rest (21). The three studies analyzing patients with Fontan circulation that included resting SpO_2_ found lower values in patients with CHD compared to the healthy controls at both LA and HA. One study found a significantly lower decrease in resting SpO_2_ for patients with CHD when moving to HA compared to controls (20), while the other two did not find any difference (19, 22). As shown in Figure 2D, the meta-analysis revealed that patients with CHD exhibited a lower decrease in SpO_2_ during peak exercise between LA and HA compared to the controls [MD: 1.22% (95% CI: 0.14–2.30)]. When the analysis was restricted to the three studies on patients with Fontan circulation, the decrease in SpO_2_ during peak exercise between LA and HA remained lower compared to the controls [MD: 1.65% (95% CI: 0.85–2.45), Supplementary Figure S1D]. One of these studies reported a SpO_2_ during peak exercise in patients with CHD at HA of 75 ± 5%.

#### 3.2.7 Symptoms

Only one study reported light symptoms of acute mountain sickness [(AMS) including dizziness and headache] in three subjects, of whom two were controls and one had CHD (21). No subjects presented with exercise-induced symptoms.

### 3.3 Risk of bias and quality assessment

**Figure 3:**
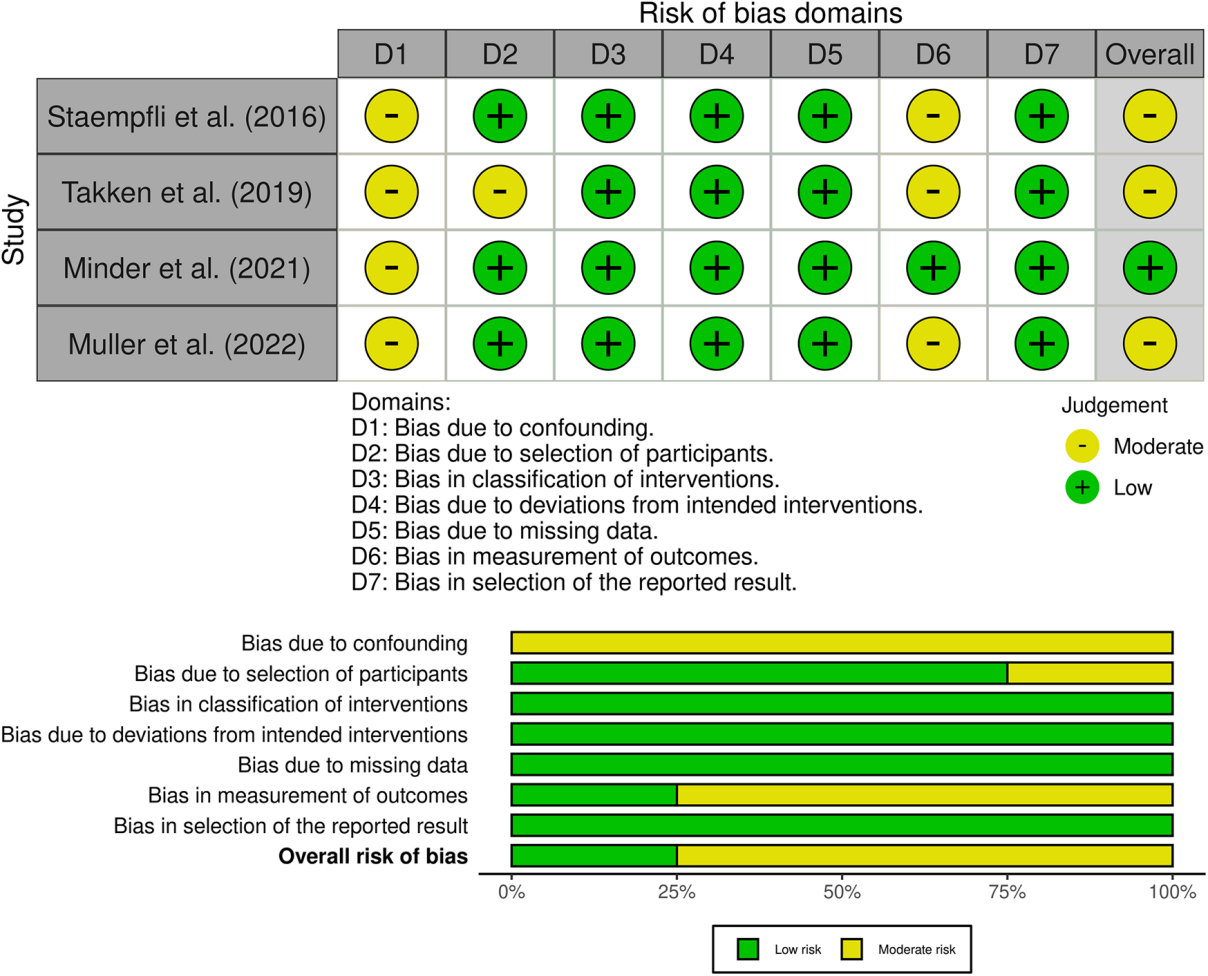
Risk of bias graph. The upper part represents the overall and single domains judgments for each study and the lower part represents the percentages of studies according to the judgments. The studies were classified as “low,” “moderate,” “serious,” or “critical” risk of bias.

The overall analysis revealed that all studies had a moderate risk of bias according to the ROBINS-I criteria (Figure 3). More specifically, they had a moderate risk of bias in the confounding domain because none of them reported the participants’ pre-existing altitude exposure and altitude of residence, which should have been reported according to the STAR data reporting guidelines for clinical HA research (23). One study had a moderate risk of selection bias due to the lack of intervention specifications for all participants (20). Two studies had a moderate risk in the outcome measurement domain due to potential systematic errors resulting from the lack of calibration of the CPET system before each test (19, 22). The remaining domains were not biased. According to the Newcastle–Ottawa Scale for case–control studies, all of these studies were rated as being of good quality (Supplementary Table S1).

## 4 Discussion

As is well-known, peak exercise performance is impaired in most patients with significant CHD both at LA and HA when compared to controls, but the results of this systematic review showed that short-term HA exposure during exercise is relatively well-tolerated by patients with CHD and has no significantly different impact on cardiorespiratory responses when corrected for their values at LA. Nevertheless, patients with Fontan circulation achieved VE/VCO_2_ slope and SpO_2_ values during exercise that warrant careful consideration due to their potential clinical relevance (15).

### 4.1 Safety of exercise during short-term high-altitude exposure in congenital heart disease

Exercise studies have demonstrated that HA affects the maximum value of each CPET parameter and, for any given work rate, HR, minute VE, and cardiac output are higher during exercise at HA compared to sea level (24, 25). The results of the included studies confirm what is already known by adding an interesting comparison between patients with CHD and healthy participants.

Resting HR increased in both groups to compensate for desaturation and maintain adequate tissue oxygen delivery at HA. The patients with CHD had a lower peak HR than the controls, and all patients who had undergone the Fontan procedure manifested chronotropic incompetence, which could be part of a physiological reaction to maintain SV and cardiac output because of reduced preload (26). However, the impact of HA was minimal on peak HR in both groups. The altitudes reached and the short-term exposure were probably not enough to justify a relevant reduction of peak HR (3, 27).

The patients with CHD had impaired exercise capacity at LA and HA as well (28). Interestingly, the loss in VO_2_ peak induced by HA exposure was similar between both groups with an even significantly higher impact of HA hypoxia on peak workload in the controls (in absolute values). A decrease of 1% in VO_2_ peak was demonstrated in the healthy individuals for each additional 100-m increase at altitudes above 1,500 m above sea level (29). This implies an expected decline of 10% in VO_2_ peak at an altitude of 2,500 m and 20% at 3,500 m, which confirms the results of the included studies for both groups. The studies indirectly investigating cardiac function showed preserved cardiac contractility, pulmonary blood flow, and SV in the patients with CHD during maximal and submaximal exercise, contradicting a possible expected decrease due to hypoxic pulmonary vasoconstriction or exercise-induced hemodynamic alterations. Hence, this may suggest that pulmonary circulation, specifically in patients with Fontan circulation, was not significantly affected by hypoxia-induced vasoconstriction, probably due to associated pulmonary vascular remodeling (30).

The efficiency of ventilation-perfusion coupling during exercise can be measured with CPET and is generally expressed with the VE/VCO_2_ slope. Studies have shown that this index is elevated in cyanotic patients with CHD, regardless of altitude (31). At HA, both groups experienced an increase in the VE/VCO_2_ slope, due in part to the physiological ventilatory response to hypoxia but also suggesting a related worsening of ventilatory efficiency, indicating possible ventilation-perfusion mismatch (32, 33). In particular, patients with CHD tend to express greater superficial ventilation with higher breathing frequency and less tidal volume response, especially at HA, which may limit oxygen uptake or carbon dioxide elimination due to more dead space ventilation (34, 35). Although the VE/VCO₂ slope modification from LA to HA was similar between the patients with CHD and the controls, the values reached by patients with Fontan circulation at HA deserve particular attention, suggesting a higher risk of impaired ventilation-perfusion coupling (33). At rest, both the patients with CHD and the controls experienced desaturation when transitioning from LA to HA. Notably, additional desaturation was observed at peak exercise in both groups, and in the three studies with patients who underwent the Fontan procedure, the decrease in SpO_2_ (%) at HA compared to LA was higher in the controls, a significant result when meta-analyzed. Several pieces of evidence confirm that patients with cyanotic CHD may be adapted to lower SpO_2_ due to the need to ensure adequate oxygen delivery to the tissues even in the presence of low arterial oxygen pressures (36). Indeed, systemic oxygen transport is not only correlated with arterial oxygen saturation because, in patients with CHD, various hematological adaptations may occur (37). For instance, they frequently present with increased hemoglobin, hematocrit, and red blood cells compared to controls, guaranteeing sufficient oxygen transport (37). Moreover, they can show decreased capillary density, diminished mitochondrial oxidative enzyme concentration, and muscle atrophy (15, 34, 37). The reduced oxidative capacity in patients with CHD results in a peripheral limitation during exercise, both at LA and at HA, similar to what has been described in healthy participants exposed to long-term HA (37). Moreover, the decreased cellular aerobic capacity and lower mitochondrial volume density in patients with CHD seem to reduce cellular oxygen demand, thus avoiding cellular dysfunction even in hypoxic conditions (5, 6).

Finally, none of the participants developed exercise-induced symptoms. Other studies have tested patients with CHD during exercise at HA, showing good tolerance, but these were excluded from this review as they did not meet the inclusion criteria. The study by Garcia et al. investigated 11 patients who had undergone the Fontan procedure and performed CPET at sea level and then following acute exposure to a simulated altitude of 3,050 m (no control group), reporting no symptoms but a reduction of 22% in peak exercise capacity (38). Short-term exposure to HA and exercise was also clinically well-tolerated by patients with Fontan circulation in the studies by Quante et al. and Härtel et al. (37, 39).

### 4.2 Assessing safety during physical activity in CHD: the role of desaturation

**Figure 4:**
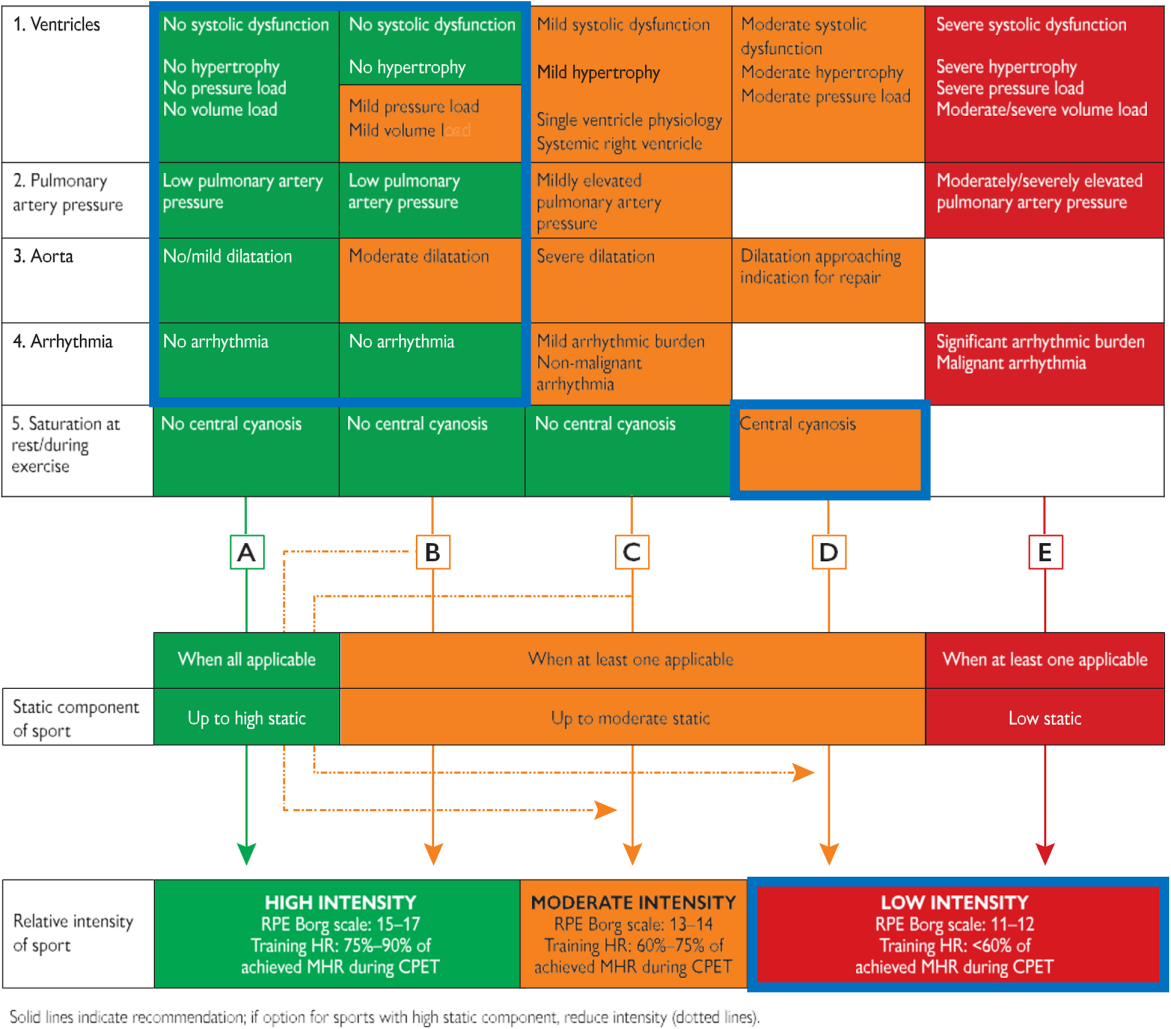
Pre-participation assessment of individuals with congenital heart disease, adapted from the ESC 2020 sports cardiology guidelines. A–E are Representative pathways linking static and intensity components for each column. After assessment of CPET and the five variables, an individual recommendation can be given (solid arrow). If a higher static level sport is chosen, then a lower intensity level is advised (dotted arrow). As marked by the blue boxes, all the patients from the included studies would belong to the D pathway (desaturation during exercise) and therefore could not perform activities beyond a low intensity. CPET, cardiopulmonary exercise test; HR, heart rate; MHR, maximum heart rate; RPE, rate of perceived exertion.

The recommendations for physical activity in adolescents and adults with CHD discuss exercise restriction for specific patients with CHD (40). These recommendations allow high intensities in the absence of ventricular dysfunction or hypertrophy, moderate to high pulmonary artery pressure, major arrhythmias and moderate or severe aortic dilatation, and central cyanosis at rest and during exercise (40). Central cyanosis can be largely excluded when transcutaneous saturations are within the range of 96%–100% at rest and during exercise. In the included and analyzed studies, all the patients with CHD were NYHA class I or II and had no major clinical features other than the presence of exercise-induced desaturation at LA, in both resting conditions, and during peak exercise at HA. Similarly, although they did not desaturate at LA, some of the healthy controls desaturated at rest and all of them did so at peak exercise intensity when exposed to HA. The lower relative reduction of peripheral oxygen saturation in the patients with CHD and the absence of any symptoms require a consideration of the role that central cyanosis may play in the pre-participation assessment of these patients. The sole presence of resting or exercise-induced desaturation would impede these patients, even in the absence of other clinical risk factors, from performing physical exercise beyond low intensity (Figure 4). Since exercise training, even at a moderate-vigorous intensity, is likely safe and beneficial for patients with clinically stable cyanotic CHD, leading to improved exercise capacity, cardiac function, and quality of life (41). Pre-participation assessment should also focus on the extent of desaturation from baseline values rather than solely on the saturation reached during peak exercise. Indeed, this review provides insight into how the role of cyanosis should be contextualized to the activities to be performed, distinguishing between resting and exercise desaturation and environmental effects.

### 4.3 Evidence for exercise recommendations in high altitude

Although the maximum intensity level reached in all included studies may allow interesting and reassuring considerations regarding acute HA exposure, concerns remain about exercise performed during prolonged exposure and at very HA (>3,500 m).

Indeed, the duration of exposure in these studies was relatively brief (less than 6 h) compared to the expected length of a real stay at HA. In contrast to acute scenarios, prolonged exposures over months or even years to moderate or HA could lead to cardiac and non-cardiac-related complications (42). However, there are conflicting results about the long-term outcomes regarding the impact of residential altitude on the risk of adverse cardiac outcomes in cyanotic patients with CHD (43–46). Moreover, based on pathophysiological considerations, it is worth mentioning that prolonged HA exposure might be associated with high-altitude pulmonary edema as has been documented in some patients with CHD and congenital vascular disease (47). The presence of underlying pulmonary hypertension predisposes individuals to the development of pulmonary edema and right ventricular dysfunction (48). Thus, elevated pulmonary vascular resistance could present specific challenges in the long term, especially for patients with Fontan circulation (49, 50). In these patients, cardiac output and stroke volume during exercise depend primarily on the venous return via the peripheral muscle pump, with minimal contribution from the ventilatory pump (51).

At HA, where fluid loss due to a diuretic response to the hypobaric hypoxic stimulus is common, a reduced venous return may further impair cardiac output (52). Although fluid balance was not assessed in the included studies, maintaining adequate hydration could be crucial for supporting hemodynamics in this population. This warrants further investigation.

For all these reasons, as expressed by a relevant expert opinion about the safety of HA travel in patients with adult CHD, pre-travel assessment should be strongly considered for physical activities at altitudes above 1,800 m in patients with moderate or high complexity CHD (53). Patients with uncomplicated simple CHD without anemia can likely travel to HA without further evaluation but should be counseled about the risks and instructed to monitor their symptoms following arrival (53).

### 4.4 Limitations and perspectives

The biggest limitation of this review undoubtedly concerns the limited number of studies and patients that can be included. Furthermore, there is a moderate risk of bias because the studies did not randomize their samples and may be biased in terms of confounding factors, selection, and measurement. However, this is a consequence of the practical difficulty in an experimental setting involving multiple LA and HA tests in such a population. This does, however, highlight the need for larger and long-term studies.

Three of the four studies only involved patients who had undergone the Fontan procedure; while these patients might be more influenced by HA compared to those with less complex CHD, the evidence for this subgroup does not appear to differ significantly from the global analysis. However, general considerations for other types of CHD are currently very limited.

None of the included studies reported the ethnicity of the participants, which limits the ability to assess the potential influence of genetic variability on their physiological responses to HA. The lack of data regarding the time elapsed since surgical treatment should be improved in future research to improve contextualization. Finally, the absence of individual raw study data precluded the expression of the impact of HA exposure on CPET variables in percentage terms. These values would have been more accurate for identifying the relative impact of the exercise at HA. However, while these were not included in the meta-analysis, the percentage differences between LA and HA CPET parameters have been reported with mean and standard deviation, when available or calculated, in Table 2.

## 5 Conclusion

Despite the limited available literature, short-term HA exposure does not appear to have a greater negative impact on exercise capacity and cardiorespiratory efficiency in patients with CHD than in healthy controls. Based on these findings, clinically stable patients with CHD could exercise at HA for short periods (up to 3,500 m and for less than 6 h). However, specific assessments to exclude exercise-related risk factors before engaging in HA activities remain recommended for patients with complex CHD. Given that LA and resting assessments cannot provide sufficient information to predict HA adaptation in patients with CHD and given the clinical value of SpO_2_ for this population, it is advisable that they always carry a pulse oximeter with them when exposed to HA, to monitor this parameter, particularly when symptoms occur. Future studies on longer-term standardized exposures to HA with a larger and more heterogeneous CHD sample are needed to obtain more data on safety and provide extensive practical recommendations for a growing population.

## Data Availability

The original contributions presented in the study are included in the article/Supplementary Material, further inquiries can be directed to the corresponding author/s.
