# Supplementary material for: Cardiopulmonary exercise response at high altitude in patients with congenital heart disease: a systematic review and meta-analysis
Source: Front Cardiovasc Med. 2024 Dec 24;11:1454680. doi: 10.3389/fcvm.2024.1454680 (PMC11703806; doi:10.3389/fcvm.2024.1454680)
Supplement: Supplementary file 1 [file Table1.pdf]

Supplementary Material

Supplementary Table 1. Newcastle-Ottawa quality assessment scale Case control studies.

		NOS Scale			
**Authors, (year)**	**Study design**	**Selection**	**Comparability**	**Exposure**	**Total (9/9)**
Staempfli et al (2016)	Case-control	✵✵✵✵	✵	✵✵	7/9 stars
Takken et al (2019)	Case-control	✵✵✵✵	✵	✵✵	7/9 stars
Minder et al (2021)	Case-control	✵✵✵✵	✵	✵✵	7/9 stars
Muller et al (2022)	Case-control	✵✵✵✵	✵	✵✵	7/9 stars

Thresholds for converting the Newcastle-Ottawa scales to quality standards (good, fair, and poor):

Good quality: 3 or 4 stars in selection domain AND 1 or 2 stars in comparability domain AND 2 or 3 stars in outcome/exposure domain

Fair quality: 2 stars in selection domain AND 1 or 2 stars in comparability domain AND 2 or 3 stars in outcome/exposure domain

Poor quality: 0 or 1 star in selection domain OR 0 stars in comparability domain OR 0 or 1 stars in outcome/exposure domain
