# Supplementary figures and images for: Cardiopulmonary exercise response at high altitude in patients with congenital heart disease: a systematic review and meta-analysis
Source: Front Cardiovasc Med. 2024 Dec 24;11:1454680. doi: 10.3389/fcvm.2024.1454680 (PMC11703806; doi:10.3389/fcvm.2024.1454680)

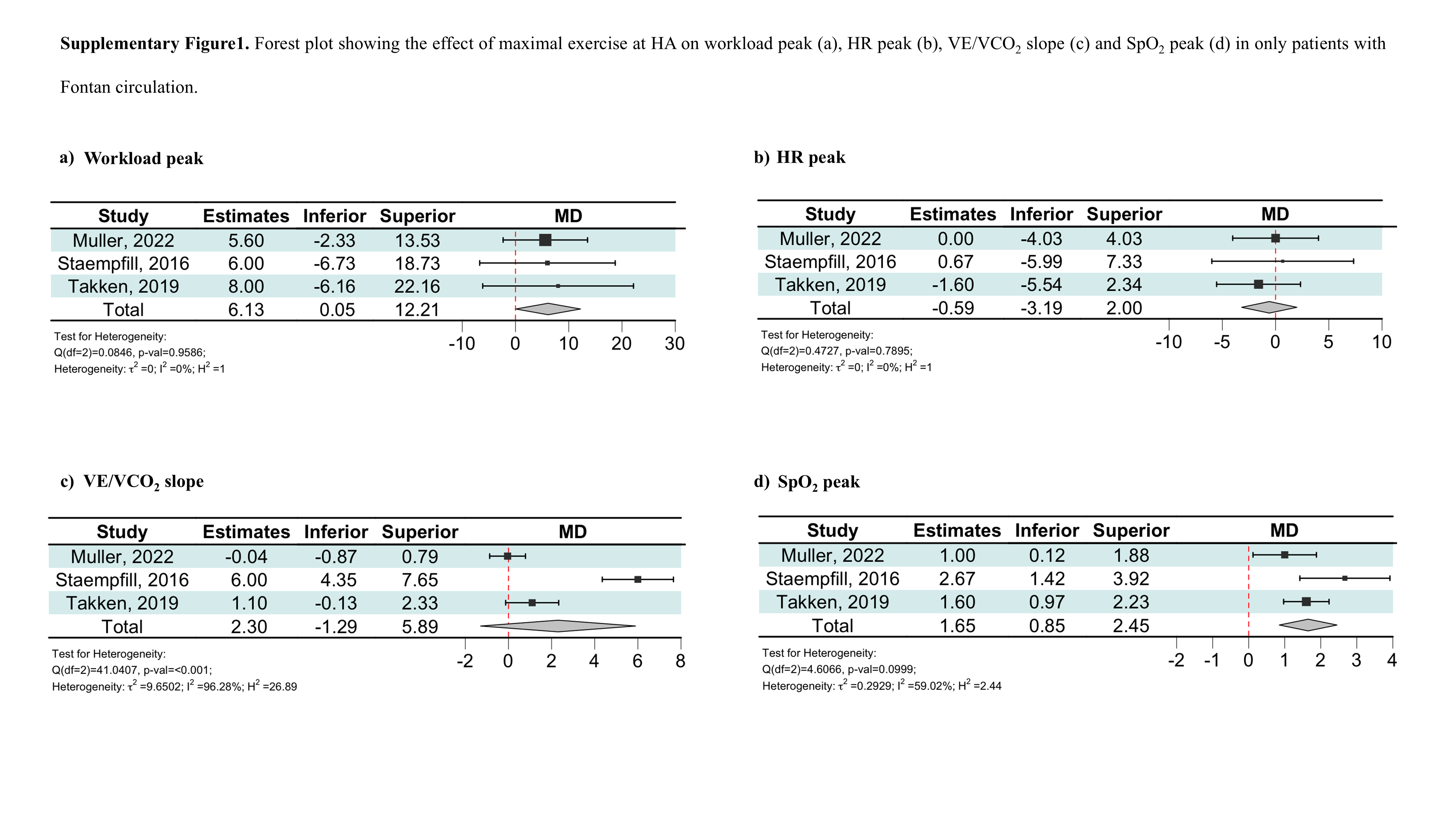

Supplement: Supplementary file 2 [file Image1.tif]
